# Simulation of bempedoic acid and ezetimibe in the lipid-lowering treatment pathway in Austria using the contemporary SANTORINI cohort of high and very high risk patients

**DOI:** 10.1007/s00508-023-02221-4

**Published:** 2023-06-08

**Authors:** Hermann Toplak, Aikaterini Bilitou, Hannes Alber, Johann Auer, Martin Clodi, Christoph Ebenbichler, Evelyn Fließer-Görzer, Carmen Gelsinger, Ursula Hanusch, Bernhard Ludvik, Thomas Maca, Andreas Schober, Reinhard Sock, Walter S. Speidl, Thomas M. Stulnig, Raimund Weitgasser, Andreas Zirlik, Marina Koch, Sebastian Wienerroither, Sorrel E. Wolowacz, Françoise Diamand, Alberico L. Catapano

**Affiliations:** 1 Department of Endocrinology and Diabetes, Clinic for Diabetology, Department of Internal Medicine, Medical University Graz, Graz, Austria; 2 Daiichi Sankyo Europe GmbH, Munich, Germany; 3 Department of Internal Medicine and Cardiology, KABEG Clinic Klagenfurt am Wörthersee, Klagenfurt, Austria; 4 Department of Internal Medicine I, Hospital St. Josef Braunau, Braunau, Austria; 5 Hospital of Internal Medicine Brüder Linz, Linz, Austria; 6 Department of Internal Medicine I, Medical University Innsbruck, Innsbruck, Austria; 7 St. Stefan, Austria; 8 Zams, Austria; 9 Centre for Clinical Studies Dr. Hanusch GmbH, Vienna, Austria; 10 Medical Department in Diabetology, Endocrinology and Nephrology and Karl Landsteiner Institute for Obesity and Metabolic Disorders, Landstraße Clinic, Vienna, Austria; 11 Evangelical Hospital Vienna, Vienna, Austria; 12 Department of Cardiology, Hospital North—Clinic Floridsdorf, Vienna, Austria; 13 Vienna, Austria; 14 Division of Cardiology, Department of Internal Medicine II, Medical University of Vienna, Vienna, Austria; 15 Department of Medicine III and Karl Landsteiner Institute for Metabolic Diseases and Nephrology, Clinic Hietzing, Vienna, Austria; 16 Department of Internal Medicine / Diabetology, Wehrle-Diakonissen Private Hospital, Salzburg, Austria; 17 Clinical Department of Cardiology, University Clinic Graz, Graz, Austria; 18 Daiichi Sankyo Austria GmbH, Vienna, Austria; 19 RTI Health Solutions, Manchester, UK; 20 Department of Pharmacological and Biomolecular Sciences, University of Milan and Multimedica IRCCS, Milan, Italy; 21 Institute for Cardiovascular and Metabolic Research (ICMR), Johannes Kepler Universität Linz (JKU Linz), Linz, Austria

**Keywords:** Lipid lowering therapy, ComBination therapy, Ezetimibe, Bempedoic acid, Cardiovascular, Atherosclerosis

## Abstract

**Objective:**

The low-density lipoprotein cholesterol goals in the 2019 European Society of Cardiology/European Atherosclerosis Society dyslipidaemia guidelines necessitate greater use of combination therapies. We describe a real-world cohort of patients in Austria and simulate the addition of oral bempedoic acid and ezetimibe to estimate the proportion of patients reaching goals.

**Methods:**

Patients at high or very high cardiovascular risk on lipid-lowering treatments (excluding proprotein convertase subtilisin/kexin type 9 inhibitors) from the Austrian cohort of the observational SANTORINI study were included using specific criteria. For patients not at their risk-based goals at baseline, addition of ezetimibe (if not already received) and subsequently bempedoic acid was simulated using a Monte Carlo simulation.

**Results:**

A cohort of patients (*N* = 144) with a mean low-density lipoprotein cholesterol of 76.4 mg/dL, with 94% (*n* = 135) on statins and 24% (*n* = 35) on ezetimibe monotherapy or in combination, were used in the simulation. Only 36% of patients were at goal (*n* = 52). Sequential simulation of ezetimibe (where applicable) and bempedoic acid increased the proportion of patients at goal to 69% (*n* = 100), with a decrease in the mean low-density lipoprotein cholesterol from 76.4 mg/dL at baseline to 57.7 mg/dL overall.

**Conclusions:**

The SANTORINI real-world data in Austria suggest that a proportion of high and very high-risk patients remain below the guideline-recommended low-density lipoprotein cholesterol goals. Optimising use of oral ezetimibe and bempedoic acid after statins in the lipid-lowering pathway could result in substantially more patients attaining low-density lipoprotein cholesterol goals, likely with additional health benefits.

## Introduction

Cardiovascular disease (CVD) remains the leading cause of death in Europe, with more than 4 million associated deaths every year [1]. Aside from increased mortality, CVD is a major cause of disability [2, 3] and reduced quality of life [4] and is associated with poor clinical outcomes [2, 3]. As well as impacting patients, CVD causes considerable economic burden with direct healthcare costs amounting to €111 billion per year in Europe [2]. Increased low-density lipoprotein cholesterol (LDL-C) is widely accepted as a proven and direct cause of atherosclerotic CVD [1, 5–7] and its major clinical sequelae [5, 8]. Each 1 mmol/L (38.67 mg/dL) LDL‑C reduction corresponds to an approximately 20% proportional risk reduction in atherosclerotic CVD events and 10% reduction in all-cause mortality [1, 5, 9], independently of the LDL‑C lowering mechanism [10–13].

Although there are well-established lipid-lowering therapies (LLTs), treatment recommendations, and risk-based goals for LDL‑C lowering in Europe [1], the population-level achievement of LDL‑C goals is limited by barriers such as statin intolerance, the limited efficacy of ezetimibe, and the cost and restricted reimbursement of proprotein convertase subtilisin/kexin type 9 (PCSK9) inhibitors [14–16]. In a European multinational (18 countries) observational study (DA VINCI), the risk-based LDL‑C goal defined by the 2019 European Society of Cardiology/European Atherosclerosis Society (ESC/EAS) guidelines was achieved in only 33% of patients prescribed LLT for primary or secondary prevention in primary or secondary care [17]. Similar findings were reported for the Austrian cohort of the DA VINCI study [18]. Many patients in routine clinical practice remain suboptimally treated and thus at increased cardiovascular (CV) risk; as a result, they need intensification of LLT for further LDL‑C lowering. Statins plus ezetimibe will only achieve LDL‑C goals on average in about 40–45% of high and very high risk patients, meaning that at least one third of these patients will require use of additional oral therapy or injectable PCSK9 inhibitor [6, 15, 17, 19–21] or inclisiran.

Bempedoic acid is a first-in-class, adenosine triphosphate citrate lyase (ACL) inhibitor, once daily, oral LDL‑C lowering treatment that can be combined with other LLTs in patients with hypercholesterolaemia (heterozygous familial and nonfamilial) or mixed dyslipidaemia who, despite current oral LLTs, are not reaching their therapeutic goals [22–25].

Bempedoic acid and its fixed-dose combination with ezetimibe were approved by the European Medicines Agency based on pivotal randomised controlled phase 3 trials in a spectrum of patients receiving maximum-tolerated statin dose (CLEAR Harmony [25], CLEAR Wisdom [23], and a fixed-dose combination study (1002FDC-053; [26])) and patients receiving no or low-dose statin (CLEAR Serenity [24] and CLEAR Tranquility [22]).

Bempedoic acid has been endorsed in consensus statements and scientific society guidance, which reinforce that it is an affordable oral treatment option that is easy to use and suitable for people who are not at goal with existing lipid-lowering treatments [6, 27–29].

This analysis aimed (1) to estimate the proportion of patients within an Austrian real-world cohort who would need additional oral LLT according to the 2019 ESC/EAS dyslipidaemia guidelines and (2) to simulate the effects of intensifying lipid-lowering therapy by addition of ezetimibe and bempedoic acid on attainment of LDL‑C goals.

## Patients, materials and methods

### SANTORINI patient cohort

The SANTORINI study (NCT04271280) is a multinational, multicentre, non-interventional, prospective, observational study, designed to describe how LLTs are used in the real world and to what extent these approaches achieve guideline recommendations [6, 30]. Patients with high or very high CV risk according to the investigator’s assessment were included in 14 European countries and were treated with LLT according to routine clinical practice. Aggregate baseline data from the Austrian patient cohort (from 17 March 2020 to 31 July 2021) of the SANTORINI observational study were used for this analysis (Table 1).

**Table 1:** Selection of eligible patients from the SANTORINI cohort for the simulation

Cohort selection (Austria)	Number of patients (%)
Overall cohort with cleaned baseline data	310 (100)
With non-missing baseline LDL‑C or recalculated with Friedewald formula	297 (95.8)
With non-missing ESC classification of risk	280 (90.3)
With non-missing intensity for statin users	275 (88.7)
Excluding patients with no LLT documented	199 (64.2)
Excluding patients receiving PCSK9 inhibitors	144 (46.5)

*ESC* European Society of Cardiology, *LDL‑C* low-density lipoprotein cholesterol, *LLT* lipid-lowering therapy, *PCSK9* proprotein convertase subtilisin/kexin type 9

Patients from the SANTORINI Austrian cohort were eligible for inclusion in the simulation (Table 1) if they were receiving any LLT, had a known statin regimen (if on statins), and had a non-missing baseline LDL‑C value (directly recorded or calculated using the Friedewald formula) [31]. Patients who at baseline were documented to be on no LLT (*n* = 76; assumed to be newly identified patients) or on a PCSK9 inhibitor (*N* = 61; assumed to have gone through treatment intensification) were excluded from the simulation. No statin intensification was simulated because it was assumed that LLT was at the maximum-tolerated regimen at baseline for those patients already on a statin or ezetimibe. Patients were stratified into high and very high CV risk groups using the risk classifications by the 2019 ESC/EAS guidelines [1].

### Simulation of the lipid-lowering therapy pathway and LDL-C reduction

**Fig. 1:**
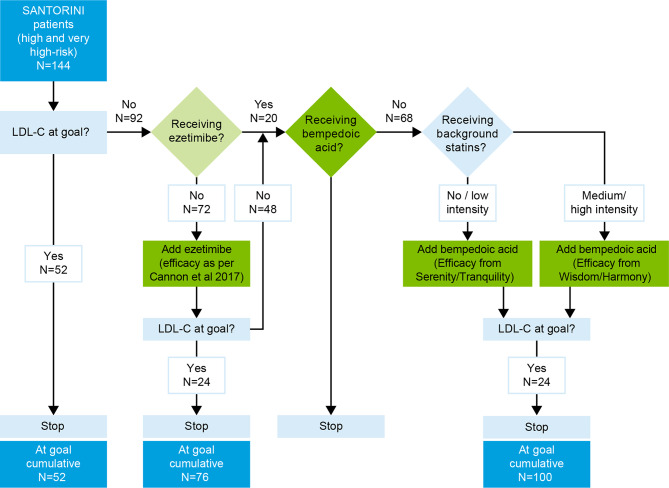
Representation of the simulation algorithm in the LLT pathway (*LDL‑C* low-density lipoprotein cholesterol, *LLT* lipid-lowering therapy)

We used a Monte Carlo simulation approach as reported previously [32–35] to simulate the addition of oral bempedoic acid after ezetimibe in the LLT pathway in the Austrian SANTORINI cohort (Fig. 1).

In the simulation, it was first determined whether the baseline LDL‑C value of patients (on an existing LLT) met their individual risk-based goal, as defined by the 2019 ESC/EAS guidelines (i.e., < 70 mg/dL or 1.8 mmol/L for those at high CV risk and < 55 mg/dL or 1.4 mmol/L for those at very high CV risk [1]). For patients not at goal, an LLT intensifying algorithm was applied (Fig. 1). The algorithm sequentially simulated the addition of ezetimibe (10 mg) and, optionally, bempedoic acid (180 mg) in case of non-achievement of LDL‑C treatment goal. The effect of the simulated treatment relied on probabilistically generated LDL-C-reduction efficacies sampled from probabilistic density functions derived from clinical trial data for each drug. The number of patients receiving treatment, the number reaching their LDL‑C goal, and the overall LDL‑C value for the patient cohort were estimated after each step of the simulation. For patients with LDL‑C levels higher than their individual goal and not already receiving ezetimibe treatment, the effect of adding ezetimibe treatment on LDL‑C was simulated. For patients still not at goal (either with ezetimibe at baseline or after addition of ezetimibe in the simulation), the effect of adding bempedoic acid treatment on LDL‑C was simulated.

The efficacy of ezetimibe was simulated in a similar way to that reported by Cannon et al. (2017), using a beta distribution with a mean LDL‑C reduction from baseline of 22.7% [36] and standard deviation (SD) of 16.5% [37]. The alpha and beta parameters of the distribution used were not reported by Cannon et al. [33]. In the present simulation, a beta distribution with alpha = 1.6 and beta = 5.4 was used, providing a reasonable approximation of the reported treatment effect (mean, 22.9%; SD, 14.8%) (Table 1).

**Fig. 2:**
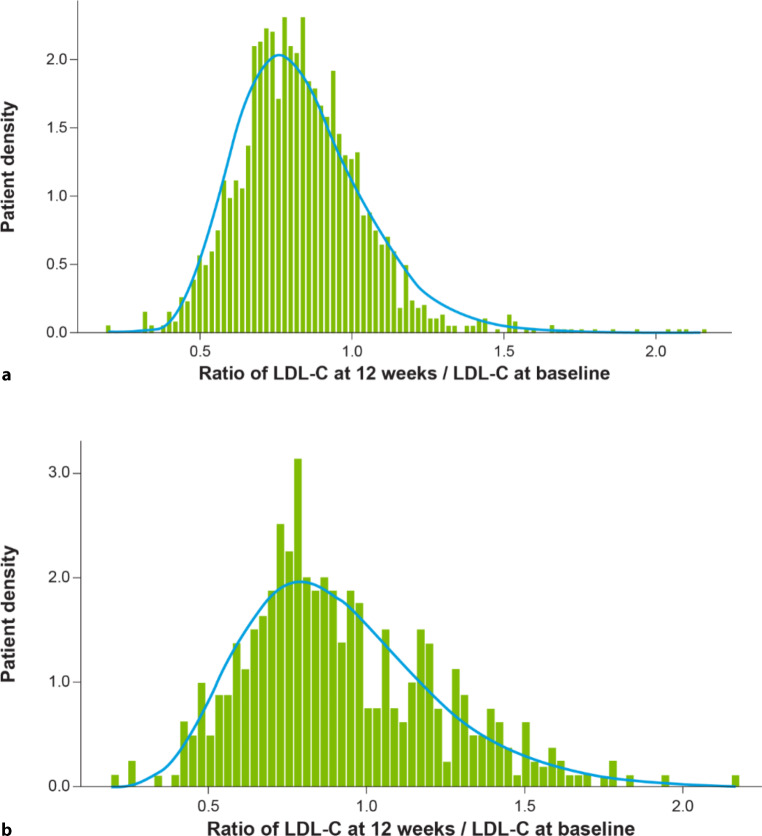
Treatment effects for bempedoic acid in patients receiving: **a** moderate or high-dose statin, Pooled Patient-Level Data from CLEAR Harmony and CLEAR Wisdom (Pool 1); *n* = 1922; **b** no or low-dose statin as background therapy, Pooled Patient-Level Data from CLEAR Serenity and CLEAR Tranquility (Pool 2); *n* = 399. *Green bars* represent the patient distribution for the ratio of LDL‑C at week 12 versus LDL‑C at baseline observed in the pooled CLEAR trial data. The *blue curves* represent the fitted lognormal distributions (*LDL‑C* low-density lipoprotein cholesterol)

The efficacy of bempedoic acid was simulated as reported previously [38] using lognormal distributions derived from the treatment effects observed in the CLEAR studies, for which patient-level data were available [38]. The lognormal distribution was selected because it allows for increases in LDL‑C level as well as decreases. For patients receiving moderate- or high-dose statin at baseline, the efficacy distribution for bempedoic acid was estimated on the basis of the pooled patient-level data from CLEAR Harmony [25] and CLEAR Wisdom (Fig. 2a, [23]). For patients receiving no or low-dose statin at baseline, the efficacy distribution for bempedoic acid was estimated on the basis of the pooled patient-level data from CLEAR Serenity [24] and CLEAR Tranquility (Fig. 2b, [22]). Table 2 presents the parameters of the distributions applied for modelling of the trial data.

**Table 2:** Distribution parameters for modelled LDL‑C reductions with ezetimibe and bempedoic acid

Treatment	Distribution	Parameters	Mean % LDL‑C reduction (SD %)^a^
Ezetimibe	Beta^b^	Alpha = 1.6 Beta = 5.4	22.9 (14.8)
Bempedoic acid (moderate- or high-dose statin background)	Lognormal	Log mean = −0.2137 Log SD = 0.2505	16.7 (21.2)^c^
Bempedoic acid (no or low-dose statin background)	Lognormal	Log mean = −0.3176 Log SD = 0.2931	24.0 (22.8)^d^

*LDL‑C* low-density lipoprotein cholesterol, *SD* standard deviation

^a^Based on LDL‑C reduction of week 12 versus baseline

^b^Adapted from [33]

^c^Modelled using patient-level data pool 1 *n* = 1922 (CLEAR Harmony and CLEAR Wisdom)

^d^Modelled using patient-level data pool 2 *n* = 399 (CLEAR Serenity and CLEAR Tranquility)

The effect of treatment on LDL‑C levels was estimated using Monte Carlo simulation with probabilistic sampling of the treatment effects; we ran 10,000 simulations of the complete set of patients. Simulations were performed using R Version 4.0.3 [39]. Variables were double programmed independently by 2 statisticians and checked, and the simulation programme was run and checked by a second statistician. After each of the 10,000 simulations, the mean LDL‑C value of the patient cohort was calculated after the addition of ezetimibe and after the addition of bempedoic acid in the simulation. The median, the 2.5% quartile, and the 97.5% quartile of these 10,000 LDL‑C means were calculated. Similarly, the number of patients at goal was estimated at the end of each simulation step, then the median, the 2.5% quartile, and the 97.5% quartile of these 10,000 LDL‑C numbers were calculated on completion of the simulation.

## Results

The baseline characteristics of high and very high-risk individuals of the Austrian SANTORINI cohort (*N* = 310) are presented in Table 3. The mean age was 65 years, 36% were female, 73% with existing cardiovascular disease (secondary prevention) and 38% diabetic. The mean LDL‑C was 93.58 mg/dL (SD 57.73), with 39.0% of patients on statin monotherapy, 12.5% on ezetimibe monotherapy or with statins, 19.6% PCSK9i (alone or in combination) and 26.1% on no lipid-lowering therapy (Table 4). From this cohort, for the purpose of the simulation, patients who were not on any LLT at baseline (*n* = 76) were excluded, as it was assumed that they were newly identified individuals of high risk needing initiation with statin or statin intolerant individuals, who would need to go through statin optimization. Patients who were already on PCSK9 inhibitors at baseline were also excluded (*n* = 61) assuming that these patients, based on prescription criteria in Austria for PCSK9 inhibitors, were on their maximum-tolerated regimen of lipid-lowering therapy and would not qualify for ezetimibe or bempedoic acid add-on; therefore, the simulation cohort used was a subset of the overall SANTORINI cohort (*n* = 144; see also Table 1).

**Table 3:** Baseline characteristics of the Austrian SANTORINI cohort overall (*N* = 310) and the simulation cohort (*N* = 144)

Characteristic	Simulation cohort (*N* = 144)	SANTORINI cohort (*N* = 310)
*Demographics*		
Age, years, mean (SD)	68.8 (10.2)	65.4 (11.4)
Female, *n* (%)	46 (31.9%)	111 (35.8%)
Diabetic, *n* (%)	74 (51.4%)	119 (38.4%)
BMI, mean (SD)	28.3 (5.0)	28.1 (4.9)
LDL‑C (SD), mg/dL, mean (SD)	76.38 (43.59)	93.58 (57.70)
*CV risk*		
High risk, *n* (%)	9 (6.3%)^a^	31 (15.1%)^b^
Very high risk, *n* (%)	135 (93.8%)^a^	169 (84.5%)^b^
*Primary prevention*	35 (24.3%)	83 (26.8%)
*Secondary prevention*	109 (75.7%)	227 (73.2%)
Myocardial infarction	38 (26.4%)	86 (27.7%)
Unstable angina	9 (6.3%)	21 (6.8%)
Stroke	15 (10.4%)	26 (8.4%)
Transient ischaemic attack	10 (6.9%)	13 (4.2%)
*Familial hypercholesterolemia*	29 (20.1%)	61 (19.7%)

Note: Data presented are mean (SD) or *n* (%) as indicated

*BMI* body mass index, *CV* cardiovascular, *EAS* European Atherosclerosis Society, *ESC* European Society for Cardiology, *LDL‑C* low-density lipoprotein cholesterol, *SD* standard deviation

^a^CV risk assessed as per ESC/EAS 2019 classification

^b^CV risk calculated for *N* = 200 patients in which the physician reported using ESC/EAS guidelines for CV risk estimation

**Table 4:** Lipid-lowering therapy at baseline for the Austrian SANTORINI cohort overall (*N* = 310) and simulation cohort (*N* = 144)

**SANTORINI cohort (*N*** **=** **310) (%)**	
*No LLT*	81 (26.1)
*Statin alone (any)*	121 (39.0)^a^
Low intensity	1 (0.3)
Medium intensity	51 (16.5)
High intensity	65 (21.0)
*Ezetimibe alone*	6 (1.9)
*Statin* *+* *ezetimibe*	33 (10.6)
*PCSK9i alone*	19 (6.1)
*PCSK9i* *+* *other LLT*	42 (13.5)
*Other LLT*	8 (2.6)
**Simulation cohort (*N*** **=** **144)**^b^ **(%)**	
*No LLT*	Excluded from cohort
*Statin (any)*	135 (93.8)
Low intensity	1 (0.7)
Moderate/high intensity	134 (93.1)
*Ezetimibe*	35 (24.3)
*PCSK9i*	Excluded from cohort

Low intensity statins include simvastatin 10 mg, pravastatin 10–20 mg, lovastatin 20 mg, fluvastatin 20–40 mg, and pitavastatin 1 mg

Moderate intensity statins include atorvastatin 10–20 mg, rosuvastatin 5–10 mg, simvastatin 20–40 mg, pravastatin 40–80 mg, lovastatin 40 mg, fluvastatin XL 80 mg, fluvastatin 40 mg twice daily, and pitavastatin 2–4 mg

High intensity statins include atorvastatin 40–80 mg and rosuvastatin 20–40 mg

*LLT* lipid-lowering therapy, *PCSK9* proprotein convertase subtilisin/kexin type 9, *SD* standard deviation

^a^121 patients on statin monotherapy, for 4 (1.29%) of whom the statin intensity was missing

^b^Patients on no LLT or on PCSK9 is were excluded from the simulation analysis. Patients on statins or ezetimibe are not necessarily on monotherapy only; they may be on combination regimens with other LLTs thus numbers may not add up to 100%

**Fig. 3:**
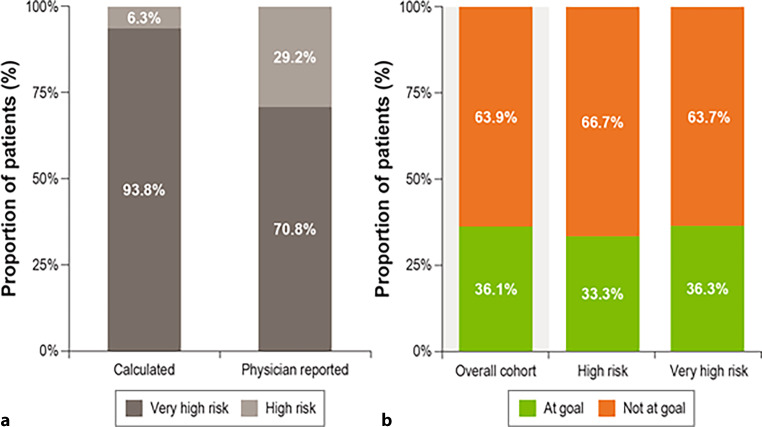
Comparison of physician-reported versus calculated CV risk using the ESC/EAS classification (**a**) and goal attainment overall and by CV risk (**b**) in the simulation cohort. CV risk was calculated as per the risk classification reported in ESC/EAS 2019 guidelines. LDL‑C goal attainment was determined according to the ESC/EAS 2019 recommendations (*CV* cardiovascular, *EAS* European Atherosclerosis Society, *ESC* European Society for Cardiology)

The mean LDL‑C of this cohort was 76.4 mg/dL, with 94% (*n* = 135) of patients on statins, the vast majority of whom were on moderate or high intensity statin (99.3%, *n* = 134); 24% (*n* = 35) were on ezetimibe, either as monotherapy or in combination (Table 4). Fifty-two patients (36.1%) had LDL‑C values meeting their risk-based ESC/EAS 2019 recommended goal. Goal attainment was similar independent of CV risk level (i.e., high or very high risk) (Fig. 3b). In addition, for those patients for whom the physician reported using the 2019 ESC/EAS guidelines for risk classification, we compared the physician-reported CV risk with the calculated risk using the patient data and medical history. Interestingly, there was an underestimation of the true CV risk for a proportion of patients (Fig. 3a).

In the simulation, out of the 92 patients not at goal, 72 were not receiving ezetimibe at baseline and thus were simulated to receive ezetimibe. A third of these patients (33%, *n* = 24 out of 72) reached their risk-based goal (Fig. 1). Another 68 patients were not at goal after ezetimibe (20 on ezetimibe at baseline and 48 still not at goal after simulation of ezetimibe) and thus were allocated bempedoic acid add-on therapy in the simulation. Addition of bempedoic acid was estimated to result in additional 35% (*n* = 24 out of 68) of patients reaching their goal.

**Fig. 4:**
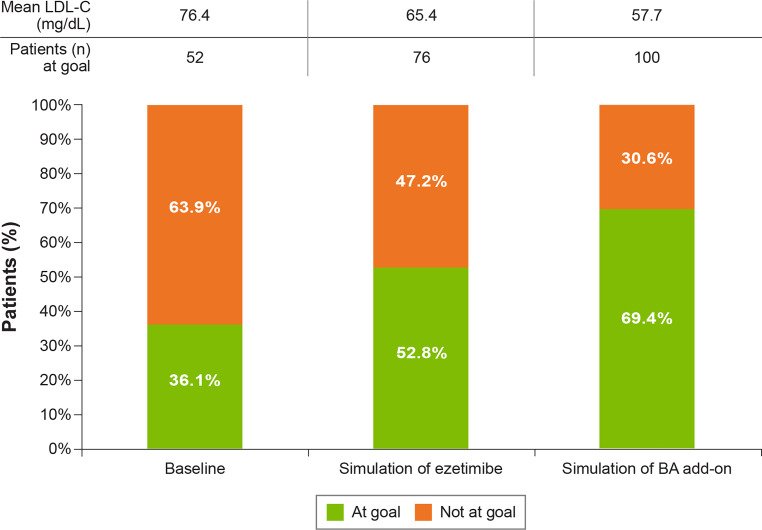
Results of the lipid-lowering therapy intensification simulation in the cohort (*N* = 144) (*BA* bempedoic acid, *LDL‑C* low-density lipoprotein cholesterol)

**Fig. 5:**
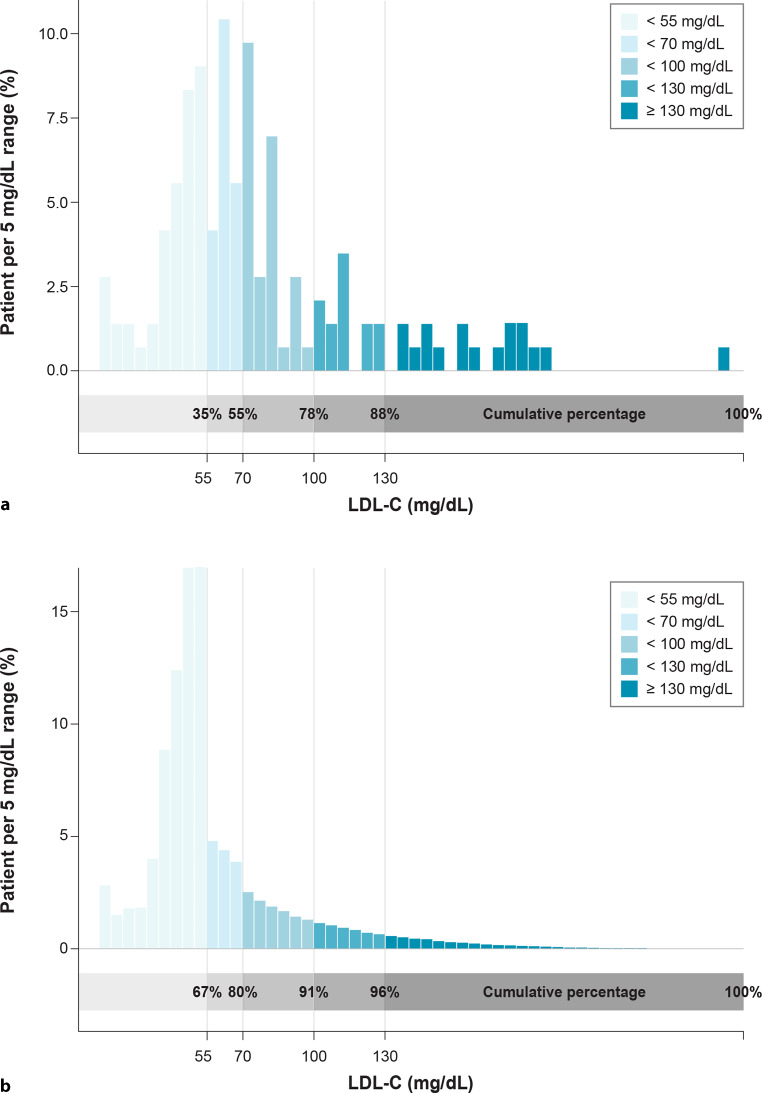
LDL‑C distribution at baseline and after lipid-lowering therapy intensification: **a** LDL‑C distribution at baseline, **b** LDL‑C distribution after the treatment simulation

Overall, the cumulative number of patients at goal in the cohort was estimated to increase from 52 (36.1%) at baseline to 76 (52.8%) and 100 (69.4%) after addition of ezetimibe and bempedoic acid, subsequently (Fig. 4). The distribution of patient LDL‑C levels at baseline and at the end of the simulation is presented in Fig. 5. The proportion of patients with LDL‑C lower than 55 and 70 mg/dL was substantially increased after the treatment simulation. For example, the proportion of patients with LDL‑C lower than 55 mg/dL almost doubled from 35–67%, and the proportion of patients with LDL‑C lower than 70 mg/dL increased from 55–80%. The mean LDL‑C for the whole cohort lowered from 76.4 mg/dL at baseline to 65.4 mg/dL and 57.7 mg/dL, after the sequential simulation of ezetimibe and bempedoic acid respectively (Fig. 4). At the end of the simulation, 74.3% of the overall cohort of 144 patients were receiving ezetimibe and 47.2% were receiving bempedoic acid.

## Discussion

In our simulation, the cumulative number of patients at their risk-based LDL‑C goal in the Austrian cohort was estimated to almost double after addition of ezetimibe and bempedoic acid, from 36% at baseline to a total of 69%. The mean absolute LDL‑C reduction for the whole cohort after this stepwise approach with oral combination treatment was **−**18.7 mg/dL, from 76.4 mg/dL at baseline to 57.7 mg/dL, with 80% of patients being at an LDL‑C level ≤ 70 mg/dL (Fig. 5).

Strengths of this analysis include (1) the use of a contemporary patient cohort from clinical centres in Austria, (2) particular attention paid to ensuring the accuracy of the LLT and LDL‑C data recorded, and (3) our model accounting for simulating a cohort to a realistic degree and considering the variability of LDL‑C reductions between different patients in response to LLT.

The baseline characteristics and treatment utilisation in the Austrian SANTORINI cohort of high and very high-risk individuals reflect contemporary data within 2 years after the 2019 ESC/EAS guideline update. Despite a high-risk patient cohort with the majority of patients with existing CV disease, treatment patterns remain similar to that reported in the previous European and Austrian DA VINCI study [17, 18], suggesting that guideline implementation in routine practice is lagging behind. CV risk underestimation and underutilisation of combination therapies as shown in our analysis may explain the low goal attainment overall. This analysis simulates how utilising the oral therapies of ezetimibe and bempedoic acid in combination with statins of any intensity would improve attainment of goals and substantially increase the proportion of patients reaching their risk-based goals before utilising PCSK9. In our analysis, a third of patients would still need further optimisation of treatment, such as addition of a PCSK9 inhibitor. Two previous simulation studies in selected German cohorts showed that addition of bempedoic acid to the LLT pathway may help reduce the need for a PCSK9 inhibitor at a population level and subsequently lower the annual overall treatment cost of therapy for healthcare systems [35, 38]. A further simulation study described the expected LDL‑C reduction after initiating bempedoic acid and estimated the potential absolute cardiovascular event risk reduction in patients with ASCVD [40]. Cardiovascular outcomes data for bempedoic acid were expected at the time of this manuscript preparation [41].

Treatment effects for bempedoic acid were simulated using lognormal distributions, which allow for both increases and reductions in LDL‑C, and closely matched the observed patient-level data from the trials. We took a conservative approach in estimating the efficacy of bempedoic acid by using all observed values of LDL‑C from the patient-level data at 12 weeks from baseline; the best-fitted distributions were used, but these potentially underestimate the treatment effect of bempedoic acid in this population. On the contrary, since no patient-level data were available for ezetimibe, we used an efficacy of 22.7% mean LDL‑C reduction simulated via a beta distribution as used by Cannon et al. [33], although its efficacy is reported between 15 and 22% due to relatively high interindividual variation [1]. Because the beta distribution is constrained to values between 0 and 100%, patients receiving ezetimibe in the simulation could only have a reduction in LDL‑C, which may not fully reflect clinical observations [42, 43]. In our analysis, we took a pragmatic approach to assume that the patients who were on a statin at baseline were at their maximum-tolerated dose, after having excluded those who were on no LLT; statin dose intensification was not further simulated. Out of the 310 patients in the SANTORINI Austrian cohort, 39% were on statin monotherapy at baseline and the majority of them were already on moderate or high intensity statin, while another 11% were on statin and ezetimibe combination therapy; this is consistent to that observed in the Austrian analysis of the DA VINCI study [18] where the vast majority of those on statin were indeed at moderate/high intensity. After excluding 26% of patients from the SANTORINI cohort who were on no LLT at baseline (assuming they were either treatment naïve, newly identified high-risk individuals, statin intolerant or not optimally treated and in need of further statin intensification), we mimic a cohort who has been maximised on their statin (and/or ezetimibe use) yet not at goal and would benefit from further oral lipid lowering therapy. It is possible that not all patients in the simulation cohort were at maximum tolerated statin dose, however, we believe that this is the closest to real-world practice.

There are certain limitations associated with this study, including the virtual basis of the analysis. But since we are extrapolating from meticulously collected real-world cohort data and since the calculations of treatment effects were performed in a conservative manner, we believe that our results are realistic and robust. The total number of patients included in the final calculation is quite low and may therefore not be representative of the Austrian high-cardiovascular risk population. Data capture was limiting and therefore we were unable to differentiate who was statin intolerant (not willing or able to tolerate statins) or treatment naïve/newly diagnosed patients and therefore these patients were excluded from the analysis. As per EAS/ESC 2019 guidelines and local treatment algorithms, the patients who were not on LLT despite having high or very high CV risk, should as a first step initiate statins, but this was out of scope for this simulation.

Furthermore, the study might be prone to selection bias because the SANTORINI study recruited patients that were deemed to be of high or very high CV risk and in need of lipid treatment. In our cohort, around 20% of individuals had HeFH which is somewhat higher than the overall proportion reported in epidemiological studies. This may be related to a selection bias since HeFH patients are most often being enrolled in registries such as the SANTORINI study and centers for lipid management. Lastly, the treatment effect of add-on LLT can only be expected if the medication is taken compliantly; in our analysis we did not account for non-adherence, neither did we account for adverse effects/safety, following similar approach to previous simulation studies.

In conclusion, optimising further the oral LLT pathway after statins by the use of bempedoic acid and ezetimibe add-on could result in substantial improvements in the number of patients reaching their LDL‑C goals.
